# Supplementary material for: Causal Relationship between Aging and Anorexia Nervosa: A White-Matter-Microstructure-Mediated Mendelian Randomization Analysis
Source: Biomedicines. 2024 Aug 16;12(8):1874. doi: 10.3390/biomedicines12081874 (PMC11351342; doi:10.3390/biomedicines12081874)
Supplement: Supplementary file 1 [file biomedicines-12-01874-s001.zip › Supplementary Figure/Figure S9/Leaveoneout plot of MR analysis for ubm-b-1454 to AN.pdf]

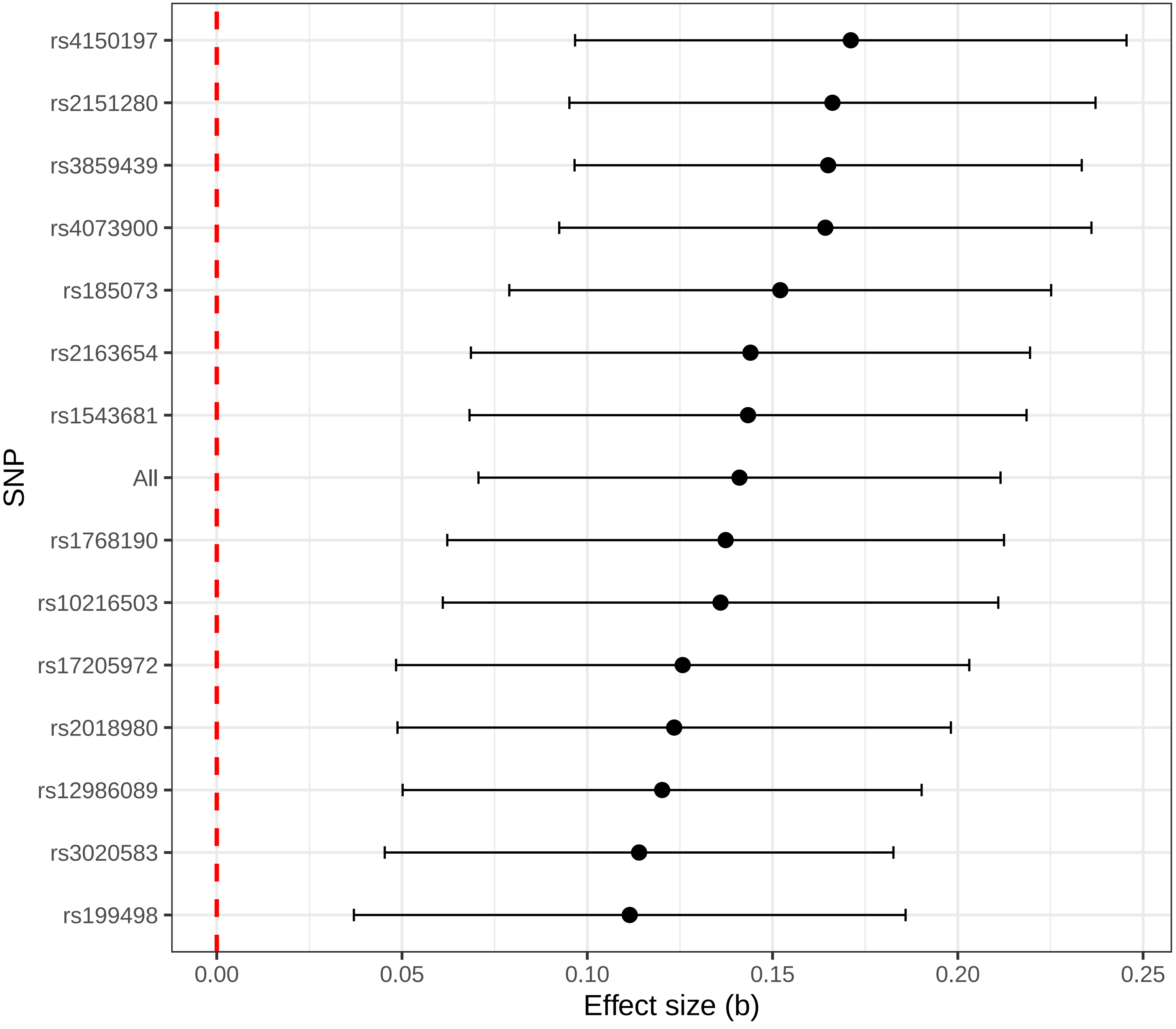
rs4150197
rs2151280
rs3859439
rs4073900
rs185073
rs2163654
rs1543681
All
rs1768190
rs10216503
rs17205972
rs2018980
rs12986089
rs3020583
rs199498
SNP
0.00
0.05
0.10
0.15
0.20
0.25
Effect size (b)
